# Delayed sarcoidosis onset mimicking mediastinal lymphoma recurrence after complete remission of diffuse large B cell lymphoma: A case report

**DOI:** 10.1111/1759-7714.13885

**Published:** 2021-02-21

**Authors:** June Hong Ahn, Min Hye Jang

**Affiliations:** ^1^ Division of Pulmonology and Allergy, Department of Internal Medicine, College of Medicine Yeungnam University and Regional Center for Respiratory Diseases, Yeungnam University Medical Center Daegu South Korea; ^2^ Department of Pathology Yeungnam University College of Medicine Daegu South Korea

**Keywords:** lymphoma, mediastinum, remission, sarcoidosis

## Abstract

Sarcoidosis‐lymphoma syndrome describes a pathological state wherein both sarcoidosis and lymphoma are present. Sarcoidosis and lymphoma may occur concurrently, or sarcoidosis may precede lymphoma. There are few reports which have previously described the temporal progression from lymphoma to sarcoidosis. Here, we present a patient with stage II diffuse large B‐cell lymphoma in the right breast. The patient achieved complete remission after chemotherapy. Five years after remission, the patient visited our clinic with newly developed enlarged mediastinal lymph nodes; lymphoma recurrence was suspected. However, mediastinal lymph node biopsy showed numerous noncaseating granulomas with no evidence of malignancy in the mediastinal lymph nodes. Consequently, a diagnosis of sarcoidosis was made. This case report highlights the need for pathological confirmation following biopsy when recurrence of lymphoma is suspected.

## Graphical abstract

We present a rare case of extremely delayed‐onset sarcoidosis mimicking mediastinal lymphoma recurrence after complete remission of diffuse large B cell lymphoma (DLBCL). Because CT and PET scans are insufficient for definitive diagnosis, pathological confirmation through biopsy is warranted when recurrence of lymphoma is suspected.

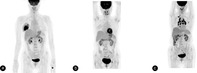

## INTRODUCTION

Sarcoidosis and lymphoma are generally considered mutually exclusive diseases. However, in 1986, Brincker coined the term “sarcoidosis‐lymphoma syndrome” (SLS) to describe their occasional association.^1^ In SLS, sarcoidosis and lymphoma may be concurrent, or sarcoidosis may precede lymphoma.^2^ There have been few reports which have previously described the progression from lymphoma to sarcoidosis.^2^, ^3^, ^4^, ^5^, ^6^, ^7^

Here, we present a case of delayed sarcoidosis onset five years after complete remission (CR) of lymphoma.

## CASE REPORT

**FIGURE 1:**
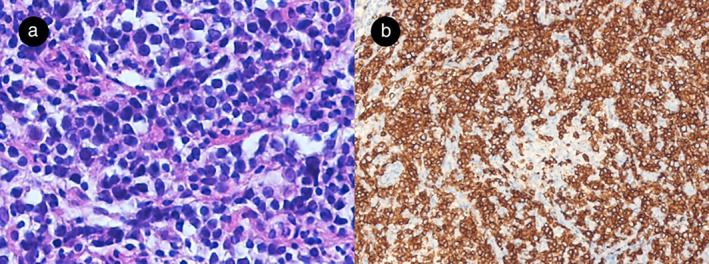
(a) Medium to large sized monomorphic malignant lymphoid cells are observed (hematoxylin & eosin [H&E] x 200). (b) The tumor cells showed CD20 positivity (x 100)

**FIGURE 2:**
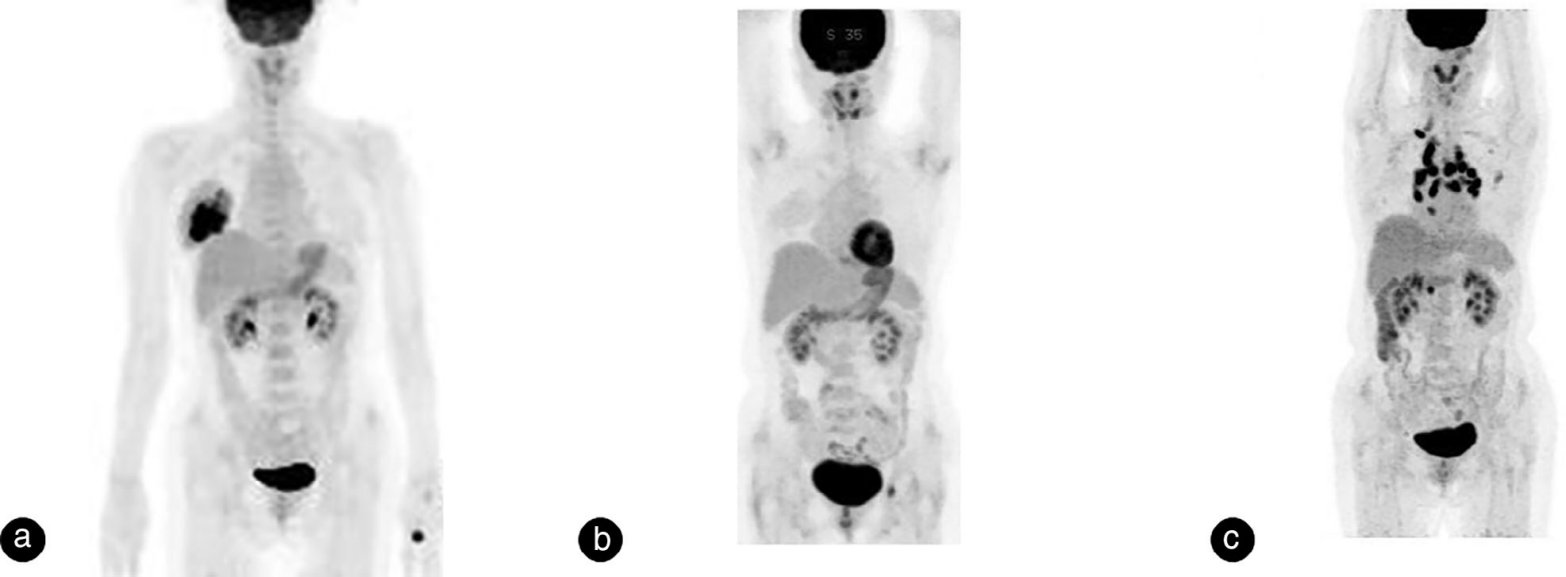
(a) Positron emission tomography (PET) scan shows a hypermetabolic mass in the right breast, and fluorodeoxyglucose uptake in the right axillary lymph node, on diagnosis of stage II diffuse large B‐cell lymphoma. (b) After six cycles of chemotherapy, PET scan shows complete metabolic response of lymphoma. (c) PET scan revealed newly developed hypermetabolic bilateral mediastinal lymph nodes

In October 2013, a 52‐year woman visited our hospital with a palpable breast mass. A right breast biopsy revealed medium to large‐sized monomorphic malignant lymphoid cells with CD 20 positivity (Figure 1). She was diagnosed with diffuse large B cell lymphoma (DLBCL) in the right breast with right axillary lymph node involvement. Bone marrow biopsy revealed no evidence of lymphoma involvement. Positron emission tomography (PET) showed abnormal fluorodeoxyglucose (FDG) uptake in the right breast and right axillary lymph node (Figure 2(a)). The patient was diagnosed with stage II disease and treated with six cycles of R‐CHOP (rituximab, cyclophosphamide, doxorubicin, vincristine, and prednisone) from November 2013 to February 2014. She achieved a CR (Figure 2(b)) and underwent follow‐up computed tomography (CT) every three months.

**FIGURE 3:**
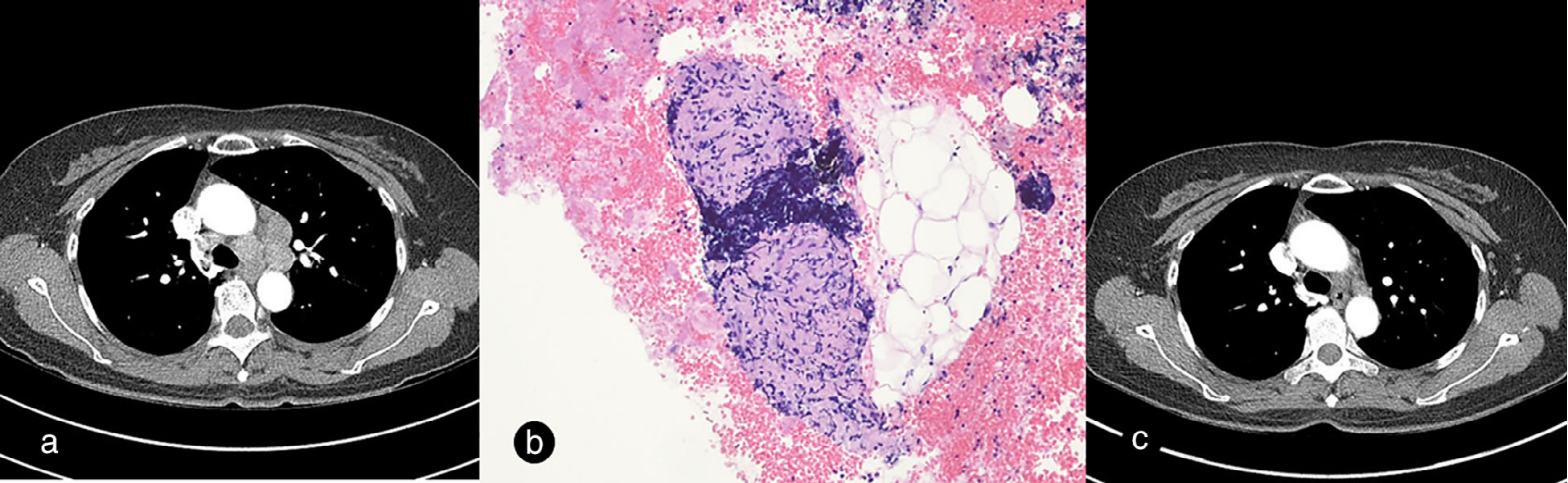
(a) Computed tomography (CT) images of enlarged mediastinal lymph nodes. (b) Numerous small and noncaseating granulomas are evident with small lymphoid cells in the background. (H&E, ×100). (c) CT scan showing the decreased size of multiple mediastinal lymph nodes

Five years after CR, the patient returned to the clinic when chest CT scan showed enlarged mediastinal lymph nodes (Figure 3(a)). Her blood test panel, including the complete blood count, liver function, renal function, and lactate dehydrogenase were normal. A PET scan revealed newly developed hypermetabolic bilateral mediastinal lymph nodes, suggestive of mediastinal lymphoma recurrence (Figure 2(c)). To confirm the diagnosis, an endobronchial ultrasound‐guided transbronchial needle aspiration (EBUS‐TBNA) of the mediastinal lymph nodes (lower paratracheal and subcarinal lymph nodes) was performed. Biopsy results showed numerous noncaseating granulomas, with no evidence of malignancy in the mediastinal lymph nodes (Figure 3(b)). Acid fast bacilli and fungi were not identified. Gomori's methenamine silver (GMS) stain and tuberculosis polymerase chain reaction (PCR) were negative. Thus, we diagnosed the patient with sarcoidosis, and used an observational treatment strategy since she had no symptoms and normal pulmonary function. Her six‐month follow‐up CT scan showed a marked decrease in the size of mediastinal lymph nodes (Figure 3(c)). At the last follow‐up, she was asymptomatic and in good health.

No ethical committee approval was required for this case study, which is in compliance with institutional and national policies. The patient consented to the publication of clinical details and images concerning the case.

## DISCUSSION

Several common features of SLS have been previously described in the literature. Overall, lymphoma is observed 5.5 times more frequently in patients with sarcoidosis than in the general population.^1^ Sarcoidosis and lymphoma usually occur concurrently, although sarcoidosis may develop prior to lymphoma; reports of lymphoma preceding sarcoidosis are rare.^2^ Typically, lymphoma follows sarcoidosis after a median of 24 months. The median age at sarcoidosis diagnosis is 10 years older for SLS patients compared with sarcoidosis patients without lymphoma. In addition, Hodgkin's lymphoma is more likely to be associated with sarcoidosis than other lymphoma types. To the best of our knowledge, this is the first case of sarcoidosis occurring more than five years after treatment for non‐Hodgkin's lymphoma.

Sarcoidosis is a systemic disease characterized by granulomatous inflammation in affected organs. The pathogenesis of pulmonary sarcoidosis involves antigen presentation followed by CD4+ activation in regional lymph nodes, and subsequent organization of macrophages into granulomas.^8^ The pathogenesis of SLS is still uncertain, but granulomatous inflammation can be considered as an aberrant cell‐mediated immune hypersensitivity reactions to tumor‐derived antigens.^9^ Typically, sarcoidosis develops in patients shortly after successful lymphoma chemotherapy. It is uncertain whether sarcoidosis development is related to an excessive immune reaction against the lymphoma cells, but this immune reaction has been associated with a good prognosis in previous studies. A sarcoid‐like reaction has also previously been reported to be associated with a better prognosis in patients with Hodgkin's lymphoma.^7^, ^10^ Delayed sarcoidosis onset after complete remission of DLBCL may be coincidental, but considering the results of previous studies, the association is meaningful.

The clinical presentation of sarcoidosis after lymphoma treatment involves incidental mediastinal or hilar lymphadenopathy with no constitutional symptoms.^7^ CT and PET scans are inadequate for distinguishing lymphoma relapse and sarcoidosis. Thus, histological confirmation is necessary.^11^

The prognosis of sarcoidosis after lymphoma is generally mild. In our case, the six‐month follow‐up CT scan showed a marked decrease in mediastinal lymph node size. This is in agreement with a previous report in which only 41% of patients required treatment, whereas 36% of patients required oral corticosteroids; 91% of those patients showed a complete clinical response.^7^

In conclusion, we present a rare case of delayed‐onset sarcoidosis mimicking mediastinal lymphoma recurrence after CR of DLBCL. Because CT and PET scans are insufficient for a definitive diagnosis, pathological confirmation through biopsy is warranted when recurrence of lymphoma is suspected. An observational treatment strategy is reasonable in asymptomatic patients with normal pulmonary function tests.

## CONFLICT OF INTEREST

The authors declare that there are no conflicts of interest.
